# Gitelman Syndrome Provisionally Diagnosed During the First Presentation of Diabetic Ketoacidosis

**DOI:** 10.7759/cureus.14253

**Published:** 2021-04-02

**Authors:** Mojgan Jalalzadeh, David Garcia Goncalves de Brito, Shobhana Chaudhari, Armeen D Poor, Donald Baumstein

**Affiliations:** 1 Internal Medicine/Nephrology, Metropolitan Hospital Center, New York Medical College, New York, USA; 2 Anesthesiology, Metropolitan Hospital Center, New York Medical College, New York, USA; 3 Internal Medicine/Geriatrics, Metropolitan Hospital Center, New York Medical College, New York, USA; 4 Internal Medicine/Pulmonary Critical Care, Metropolitan Hospital Center, New York Medical College, New York, USA

**Keywords:** diabetic ketoacidosis, gitelman syndrome, metabolic alkalosis, hypokalemia, hypomagnesemia

## Abstract

Gitelman syndrome (GS) is an autosomal recessive disease characterized by hypokalemia, hypomagnesemia, metabolic alkalosis, and hypocalciuria. It is caused by mutations in gene SLC12A3 (located in chromosome 16q) encoding NaCl cotransporter. GS is usually asymptomatic for several years and is diagnosed in late childhood or adulthood. The association between GS and diabetic ketoacidosis (DKA) is rare. We present a case of a 25-year-old man with newly diagnosed diabetes mellitus and DKA with profound hypokalemia and hypomagnesemia who was provisionally found to have GS.

## Introduction

Gitelman syndrome (GS) is an autosomal recessive disease. The prevalence of GS has been estimated to be 1-10 in 40,000 [1]. The main clinical manifestations of GS are hypokalemia, metabolic alkalosis, hypomagnesemia, hypocalciuria, normal or low blood pressure and tetany [2]. Hypomagnesemia and hypocalcuria are characteristic of GS [3].

GS is caused by mutations in gene SLC12A3 (located in chromosome 16q) encoding NaCl cotransporter (NCCT), which is expressed in the apical membrane of cells along the distal convoluted tubule [4]. The tubular defects in sodium chloride transport result in a thiazide-like effect leading to volume contraction and activation of the renin-angiotensin-aldosterone system, ultimately causing metabolic alkalosis and hypokalemia [5].

GS is usually asymptomatic for several years and is diagnosed in late childhood or adulthood. When symptomatic, clinical manifestations include cramps of the arms and legs, fatigue, tetany polyuria and nocturia that are due to loss of magnesium and potassium by the kidneys. Chronic hypokalemia is one of the causes of nephrogenic diabetes insipidus and polydipsia. Chondrocalcinosis may occur due to severe hypomagnesemia [6]. Despite hyperaldosteronism, patients tend to have normal or low blood pressure, which is explained by the vascular response to prostaglandins.

The presence of both hypocalcuria and hypomagnesemia is highly predicative of the clinical diagnosis of GS, but confirmation of suspected GS rests on genetic testing [1]. Therapeutic approaches to GS include potassium and magnesium supplements, prostaglandin synthesis inhibitors (nonsteroidal anti-inflammatory drugs), aldosterone antagonists, and angiotensin-converting enzyme inhibitors [7]. The prognosis of this syndrome with treatment is excellent. However, some patients develop diarrhea because of high doses of oral magnesium that increases gastrointestinal loss of magnesium. It should be noted that the association between GS and type 1 diabetes mellitus has not been frequently reported.

In this report, we describe the findings and treatment of a young patient who presented with a new onset of diabetic ketoacidosis (DKA), who, after managing hyperglycemia and hyperosmolarity, still had profound hypokalemia and hypomagnesemia and was provisionally diagnosed with GS.

## Case presentation

A 25-year-old White male with no prior available laboratory tests and no significant past medical or psychiatric history presented to the emergency room complaining of abdominal pain, nausea, vomiting, weight loss, profound fatigue associated with polyuria, and dehydration. He had a positive family history of type 1 diabetes from his mother. During physical examination, he was alert, afebrile, with blood pressure of 126/84 mmHg, pulse rate of 107, respiratory rate 18, with dry mucous membranes and reduced skin turgor.

**Figure 1:**
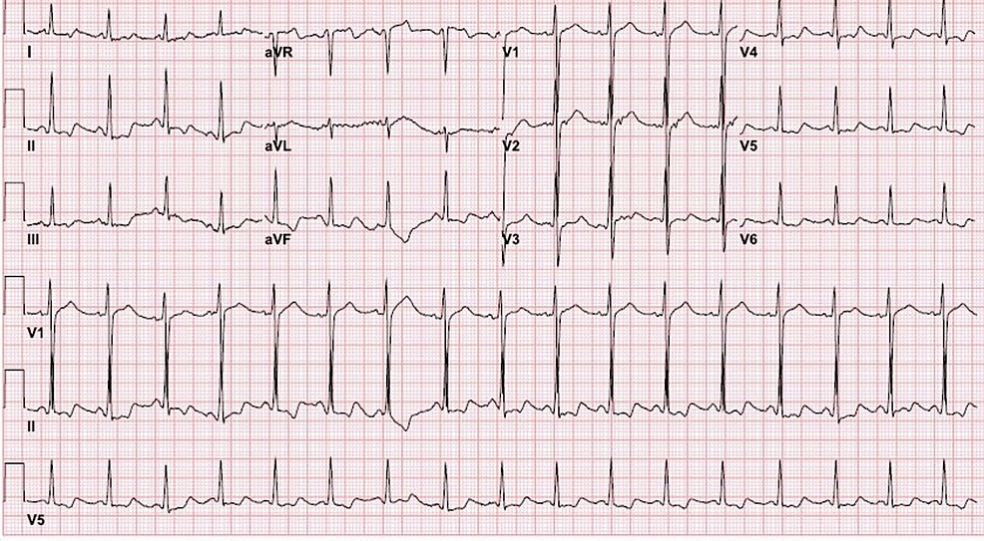
Electrocardiogram showing sinus tachycardia and prolonged QT interval (QT/QTc 406/529 ms)

His lab tests were notable for plasma glucose 479 mg/dL with large amounts of serum and urine ketones, pH 7.15, anion gap 36, lactic acid 2.7 mmol/L, calculated serum osmolality 288 mOsm/L, serum sodium 129 mEq/L, potassium 3.2 mEq/L, bicarbonate 9 mmol/L, chloride 84 mEq/L, calcium 9.4 mg/dL, phosphorous 2.6 mg/dL, magnesium 1.3 mg/dL, HbA1C 14.4%, urine glucose > 1000 mg/dL and urine toxicology negative. Electrocardiogram demonstrated sinus tachycardia and prolonged QT interval (Figure 1). He was diagnosed with DKA and was admitted to the intensive care unit.

Intravenous insulin was started after magnesium, potassium, and phosphorus replacement. Intravenous insulin was stopped, or dosage reduced several times due to the severity of hypokalemia. A total of 400-450 mEq per day of potassium chloride was administered intravenously and orally during the first three days. Also, total magnesium sulfate 6-8 g was given daily for three days. In the first four days, his daily urine output ranged from 5.5 to 6.5 L. This was repleted with oral and intravenous fluids. Hyperglycemia, ketonemia and anion gap metabolic acidosis resolved after four days.

On day 5, his clinical condition improved, and acidosis resolved. His biochemical tests showed metabolic alkalosis with bicarbonate level of 38 mmol/L, and the patient remained alkalotic without any signs of dehydration. He had no vomiting and did not receive sodium bicarbonate or diuretics throughout hospitalization. He was not on any other medications.

Furthermore, despite aggressive repletion of potassium and magnesium, the patient remained hypokalemic and hypomagnesemic. He required 40-80 mEq of potassium chloride and 1 g of oral magnesium daily after acidosis and polyuria resolved to bring the serum potassium and magnesium concentrations to the normal level.

On day 8, while the patient was being discharged from the hospital, his urinary electrolytes and serum electrolytes were checked. The lab results showed potassium 4.5 mEq/L, magnesium 1.5 mg/dL, calcium 9.2 mg/dL, bicarbonate 33 mg/dL, and serum creatinine 0.6 mg/dL. The urine random studies revealed sodium 32 mEq/L, chloride 54 mEq/L, potassium 79 mEq/L, magnesium 8.5 mg/dL, calcium 6.5 mg/dL, phosphorus 48.8 mg/dL and creatinine 60.3 mg/dL.

In this case, the management of ketoacidosis required high doses of intravenous potassium chloride to obtain normal serum potassium levels. After the management of DKA, the patient showed continued hypokalemia and hypomagnesemia with a new metabolic alkalosis while urine chloride was more than 40 mEq/L.

He was discharged with Levemir® insulin, lispro insulin, oral magnesium gluconate and potassium chloride. He was also advised to go to the nephrology clinic for follow-up.

Seven weeks after discharge, his repeated random urine study revealed sodium 69 mEq/L, chloride 125 mEq/L, potassium 79 mEq/L, magnesium 13.8 mg/dL, calcium 3.5 mg/dL, phosphorus 23.8 mg/dL and creatinine 162 mg/dL. Unfortunately, there were no serum electrolytes obtained since hospitalization.

## Discussion

Gitelman syndrome is a rare disease. In the Kidney Disease: Improving Global Outcomes (KDIGO) Controversies Conference, the following criteria were proposed for suspecting GS: chronic hypokalemia (< 3.5 mmol/L) with renal potassium wasting, metabolic alkalosis, hypomagnesemia (<1.70 mg/dL) with inappropriate magnesium wasting, hypocalcuria, high plasma renin activity, fractional excretion of chloride > 0.5%, low or normal-low blood pressure and normal renal ultrasound [1].

In addition, one needs to exclude other differential diagnoses of normotensive hypokalemic metabolic alkalosis such as diuretic and laxative use, vomiting and a rare complication of cisplatin use. In our patient, we excluded other diagnoses by history as outlined in the Case Presentation. In addition, his high urine chloride, 54 mEq/L on day 8 and 125 mEq/L seven weeks after hospitalization, rules out surreptitious vomiting.

He manifested all the criteria that were available for the assessment in a patient suspected of having the diagnosis of GS. On day 8 of hospitalization, four days after the resolution of his DKA, he had a normotensive metabolic alkalosis. On day 8, his spot urine calcium (mg/dL) to urine creatinine (mg/dL) ratio was calcium 6.5 (mg/dL):creatinine 60.3 (mg/dL) equaling 0.108, and seven weeks later was 3.5:162 (mg/dL) or 0.022. In a recent series of 29 adults with genetically confirmed Gitelman syndrome, the spot urine calcium/creatinine ranged from 0 to 0.14 [8]. Our patient falls in this range on both spot samples. Bartter syndrome has many overlapping manifestations. It is another genetic renal tubular disorder that causes salt wasting but through a loop diuretic-like effect. It yields an elevated urine chloride like in GS, but in Bartter syndrome, urine calcium is normal or elevated.

Other lab studies provide further support for the diagnosis of GS. On day 8 of hospitalization, his magnesium was 1.5 mg/dL and his fractional excretion of magnesium was 5.6%, above the threshold of 4% outlined in the KDIGO Conference report. This represents inappropriate magnesium wasting. In the setting of hypokalemia, appropriate urine potassium retention would be indicated by a urine potassium level <25 mEq/L or urine potassium-to-creatinine ratio <15 mEq/g. In this case, the urine K was 79 mEq/L and urine potassium-to-creatinine ratio was 131.6 mEq/g. His receiving potassium and magnesium replacement complicated this assessment of potassium and magnesium wasting; however, his massive potassium and magnesium requirements and other above-noted aspects support the diagnosis of GS. He did not have a plasma renin activity or renal sonogram, which are the remaining criteria to suggest a GS diagnosis.

In our literature review, diabetic ketoacidosis in GS was found to be reported in only two cases: a 14-year-old patient [9] who was diagnosed with GS before the onset of diabetes and a 10-year-old patient who was first diagnosed with DKA and then developed GS one month later [10]. In our patient, DKA occurred as the first manifestation of diabetes. The profound hypokalemia, hypomagnesemia and expression of metabolic alkalosis after DKA treatment allowed us to identify the presence of GS, which was not revealed on initial presentation.

DKA has been described as a biochemical triad of hyperglycemia, ketonemia, and metabolic acidosis [11]. Profound hypokalemia is an unusual early manifestation in patients with DKA and indicates severe whole-body potassium deficiency. In such cases, potassium repletion should be undertaken during correction of other metabolic abnormalities, including hyperglycemia and hyperosmolality. Intravenous insulin should be avoided unless the serum potassium concentration is above 3.3 mEq/L [12].

The presence of congenital tubulopathy as GS with metabolic alkalosis can mask acute DKA in the initial presentation [9]. In addition, DKA is characterized by hypokalemia because of a significant excretion of renal salts, and subsequent administration of insulin worsens the hypokalemia. The concomitant presence of DKA with GS, which is characterized by hypokalemia, can make management of DKA very difficult.

Hypomagnesemia in GS is assumed to be secondary to a primary defect of the NCCT, possibly due to the down-regulation of transient receptor potential magnesium 6 and 7 (TRPM-6, TRPM-7) in the distal convoluted tubule [13]. Magnesium depletion releases inhibition of renal outer medullary potassium channels and leads to increased potassium excretion [13].

Amiloride is a selective blocker of the epithelial sodium channel (ENaC) [14]. ENaC blockers are thought to increase magnesium absorption based on their alteration of transepithelial voltage. When ENaC is blocked, cells in the distal nephron are unable to absorb sodium, which creates a positive luminal and negative intracellular charge. This electrical gradient favors magnesium absorption through TRPM-6 and TRPM-7 channels.

The patient according to above findings is considered to have a very high clinical suspicion for GS. Genetic testing is becoming increasingly available and would provide confirmation of this diagnosis. Based on his follow-up laboratory results, treatment would include ad lib salt consumption, oral potassium and/or magnesium supplementation and consideration of amiloride or eplerenone.

## Conclusions

In cases with diabetes and DKA, when the management of hypokalemia is difficult, it is important to consider the diagnosis of some tubulopathies such as GS. In addition to hypokalemia, inappropriate renal magnesium excretion in the setting of hypomagnesemia, metabolic alkalosis, and low urine calcium are consistent with a diagnosis of GS.
